# Impact of Bariatric Surgery Type on Adipokines, Myokines, and Hepatokines: An 18-Month Prospective, Observational, Open-Label Study

**DOI:** 10.33549/physiolres.935756

**Published:** 2025-12-01

**Authors:** Zdeněk ŠVAGERA, Pavol HOLÉCZY, Karolína JANOCHOVÁ, Marek BUŽGA

**Affiliations:** 1 Institute of Laboratory Medicine, Faculty of Medicine, University of Ostrava, Ostrava, Czech Republic; 2 Institute of Laboratory Medicine, Department of Clinical Biochemistry, University Hospital Ostrava, Ostrava, Czech Republic; 3 Department of Surgery, AGEL Ostrava-Vitkovice Hospital, Ostrava, Czech Republic; 4 Department of Surgical Studies, Faculty of Medicine, University of Ostrava, Ostrava, Czech Republic; 5 Department of Physiology and Pathophysiology, Faculty of Medicine, University of Ostrava, Ostrava, Czech Republic

**Keywords:** Bariatric surgery, Adipokines, FGF19, FGF21, ANGPTL

## Abstract

Bariatric surgery is the most effective way to treat obesity and improves obesity-related comorbidities. Laparoscopic sleeve gastrectomy (LSG) is one of several standard procedures, laparoscopic greater curvature plication (LGCP) is a relatively alternative bariatric technique, and Roux-en-Y gastric bypass (RYGB) is the gold standard of bariatric surgical procedures. The study included 95 patients who underwent three types of bariatric surgery. 48 of the subjects (28 women, 20 men) underwent LSG, 35 of the patients (21 women, 14 men) underwent LGCP and 12 of the subjects (8 women, 4 men) underwent RYGB. Anthropometry and biochemical parameters (glucose, glycated hemoglobin, cholesterol, HDL and LDL cholesterol, triglycerides, adiponectin, leptin, ANGPTL3, ANGPTL4, ApoD, ApoE, FGF19, and FGF21) were determined before and after 3, 6, 12 and 18 months of surgeries. All types of bariatric surgeries markedly decreased body weight, BMI, and percentage of body fat. The surgical procedures resulted in a decrease in mean fasting glucose, triglycerides, glycated hemoglobin concentrations and leptin concentrations in blood serum. On the other hand, plasma concentrations of adiponectin increased significantly. Different results were observed in serum ANGPTL3, ANGPTL4, ApoD, ApoE, FGF19, and FGF21 levels after all surgeries. All three types of bariatric surgery resulted in significant weight loss and changes in the levels of the measured parameters.

## Introduction

One of the most significant challenges of current medicine is the increasing prevalence of obesity worldwide, which is accompanied by a wide range of chronic complications and increased mortality. Globally, according to the World Health Organization (WHO) in 2022, more than 2.5 billion adults were overweight (43 %) or obese (16 %). The prevalence of overweight varied by region, from 31 % in the WHO South-East Asia Region and the African Region to 67 % in the Region of the Americas [1]. The best way to reduce body weight and fat mass is by changing a lifestyle focused on balanced dietary intake and increasing physical activity. Unfortunately, this approach is not very successful. A possible solution is the use of metabolic and bariatric surgery, which has been proven to be the most effective way of treatment for morbidly obese patients with excess weight and related comorbidities [2]. Strong evidence has revealed that, in addition to weight loss, bariatric surgery further improves type 2 diabetes, hypertension, and dyslipidemia [3]. Sleeve gastrectomy (LSG) is a restrictive form of bariatric surgery. The principle of this method is to cut the bottom and the large curvature of the stomach and suture the rest of the stomach into the “sleeve” with a capacity of 60–180 ml [4]. Laparoscopic greater curvature plication (LGCP) is also a restrictive method that reduces gastric volume by plication in the region of greater curvature. The main difference between LGCP and LSG is that LGCP is a reversible technique without the use of gastrectomy [5]. Roux-en-Y Gastric Bypass (RYGB) is restrictive, malabsorption and irreversible type of bariatric surgery and it is considered by many surgeons as the “gold standard” procedure and, together with LSG, account for approximately 90 % of all operations performed worldwide [6]. RYGB includes the creation of a small proximal stomach of about 15–20 ml and bypass of the duodenum and proximal jejunum from the transit of food. The rest of the stomach is not removed, but it is completely interrupted and disconnects from the lower part. Due to the reduced intestinal length where digestion and absorption occur, RYGB is quite often accompanied by a deficiency of some vitamins and minerals (vitamin B12, vitamin D, iron, calcium) [7]. Therefore, it is necessary to monitor the patient during the postoperative period and regularly control the blood levels of these substances and compensate for any deficiencies [8]. Bariatric surgery results in long-term success in more than 80 % of patients [9]. The long-term efficacy of bariatric surgery treatment depends only on patients, if they attend regular check-ups, follow dietary recommendations, and develop adequate physical activity [10]. Like all surgical procedures, bariatric surgery carries its own risk [11].

In recent years, attention has also been focused on the metabolic effects of individual adipokines, myokines, and hepatokines after bariatric surgery [12]. It has been suggested that fibroblast growth factor 19 (FGF19) as a hepatokine representative and fibroblast growth factor 21 (FGF21) as a myokine could potentially offer a positive therapeutic benefit in the treatment of obesity and obesity-related pathologies [12]. These metabolic regulators have been found to improve obesity, insulin resistance, and dyslipidemia [13,14]. In lipid metabolism, angiopoietin-like peptide 3 (ANGPTL3) and angiopoietin-like peptide 4 (ANGPTL4) play an important role in the regulation of lipoprotein lipase activity (LPL). It is evident that ANGPTL4 exerts a marked decrease in circulating triglyceride levels by inhibiting LPL, predominantly in tissues, while ANGPTL3 functions systemically within the bloodstream. It has been demonstrated that these proteins possess the capacity to reduce LDL-C and other lipoproteins, a process that occurs independently of LDL-receptor activity [15]. Furthermore, the FDA has approved the use of a monoclonal antibody directed against ANGPTL3 for the treatment of patients suffering from homozygous familial hypercholesterolemia in February 2021 [16].

For this reason, we decided to study the effect of selected parameters, adipocytokines (adiponectin, leptin), hepatokine (FGF19), myokine (FGF21), apolipoprotein D (ApoD), apolipoprotein E (ApoE), angiopoietin like peptide 3 (ANGPTL3), angiopoietin like peptide 4 (ANGPTL4) with respect to the type of surgery used.

## Methods

### Patients (Subject characteristics)

A total of 95 subjects with a mean age of 45.1±10.0 and predominantly exceeding the BMI values 40 kg/m^2^ underwent a bariatric surgery operation. 48 of the subjects (28 women, 20 men) underwent laparoscopic sleeve gastrectomy (LSG), 35 of the patients (21 women, 14 men) underwent laparoscopic greater curvature plication (LGCP), and 12 of the subjects (8 women, 4 men) underwent Roux-en-Y gastric bypass operation (RYGB). The study had a prospective, observational and open-label design (ClinicalTrials.gov registration: NCT02893891). The study was approved by the Ethics Committee of the Faculty of Medicine of the University of Ostrava, Czech Republic, according to the ethical standards of the Declaration of Helsinki of 1975, as revised in 2000. The subjects were patients of the bariatric unit, Department of Surgery of the Vitkovice Hospital in Ostrava, Czech Republic. The selection of patients for surgical treatment of obesity was carried out in accordance with the guidelines of the International Federation for Surgery and Metabolic Disorders (IFSO).

### Bariatric surgery procedures

#### Laparoscopic Sleeve Gastrectomy (LSG)

Performed under general anesthesia. The greater curvature is mobilized and the stomach is longitudinally resected with a stapler. No bougie or staple reinforcement used. Specimen removed; drain placed if needed. Skin closed to finish.

#### Laparoscopic Greater Curvature Plication (LGCP)

Laparoscopic with five ports. The greater curvature is mobilized, preserving gastric veins. Stomach folded with non-absorbable sutures. No antibiotics given. Post-op anti-nausea and reflux meds administered.

#### Roux-en-Y Gastric Bypass (RYGB)

Laparoscopic with five ports. Small gastric pouch created. Gastrojejunostomy formed after measuring alimentary limb. Early oral fluids and mobilization. Single-dose antibiotic prophylaxis used. Discharge around day four.

### Anthropometric evaluation

A week before the planned intervention, body composition was assessed in all probands using the dual energy X-ray absorptiometry (DXA) method (Discovery A; Hologic, Waltham, MA, USA). The following parameters were monitored: weight (kg) fat (%) and lean body mass (LBM; kg). Height was measured in centimeters and weight in kilograms using a standard scale. The scale was calibrated regularly according to the standard procedures of the site, with the same scale used for all measurements. Weights were recorded for individuals wearing undergarments only, with jewellery and shoes removed. All parameters were compared with baseline data within the group and then between the groups. The densitometer was calibrated according to the manufacturer’s recommendations, and the precision error was established.

### Biochemical assessment

The patient’s blood draws were performed in the morning after fasting overnight a week before the planned procedure and then 3, 6, 12 and 18 months after the bariatric surgery operation. These blood samples, except for glycated hemoglobin, were centrifuged at 2500× g for 6 min at 4 °C. Serum concentrations of glucose, triacylglycerols (TG), high-density lipoprotein (HDL) and low-density lipoprotein (LDL) cholesterol were determined by chemical analyzer (AU5420, Beckman Coulter, Inc., USA). Glycated hemoglobin (HbA1c) was measured by HPLC (Tosoh G8, Tosoh Bioscience, Inc., CA, USA). Serum samples were aliquoted and stored at −80 °C until analysis.

Serum levels of Apo D, Apo E, ANGPTL3, ANGPTL4, adiponectin, and leptin were analyzed by the ELISA method using sandwich sets (Biovendor, Laboratorni medicina, Brno, Czech Republic) on ELISA analyzer (DSX, Dynex Technologies, Chantilly, VA, USA). FGF19 and FGF21 were analyzed by a multiplex assay performed by Biovendor, Laboratorni medicina, Brno, Czech Republic using barcoded magnetic beads and performed on Biocode 100A (Applied Biocode, USA).

#### Statistical analysis

Data analysis was performed using basic descriptive statistics (mean, standard deviation). On the basis of the Shapiro-Wilk normality test, we compared the parameters by means of the two-sample *t*-test, the two-sample Wilcoxon rank sum test (Mann-Whitney). The paired t-test and Wilcoxon rank sum test were used to assess parameter changes over time vs. baseline. Change-scores (Δ) as pre-operative minus post-operative values at 3, 6, 12, and 18 months were computed. Within each surgical group (LSG, LGCP, RYGB) and time point, we assessed associations between Δ adipokines/myokines/hepatokines and Δ anthropometric/metabolic parameters (Weight, BMI, % Fat, Glucose, HbA1c, Chol, TG, HDL, LDL) using Spearman correlation with 95 % CIs (Fisher’s z). P-values were FDR-adjusted (Benjamini-Hochberg) within each (group × time) block. Independent associations were evaluated using robust OLS (HC3) with ΔBMI (or ΔFat), ΔTG, ΔGlucose, ΔHbA1c, and the baseline biomarker value as predictors. Statistical tests were evaluated at the 5 % significance level with data processed using SPSS, version 24.

## Results

Tables 1–4 present a comprehensive summary of all anthropometric and biochemical parameters. The following discussion will address individual groups of analytes.

### Anthropometric parameters

All surgical groups demonstrated significant weight loss and reduction in BMI during the 18-month follow-up. The most pronounced decrease in body weight was observed after RYGB, while LGCP achieved the greatest reduction in body fat percentage. At 18 months, fat mass (DXA) declined significantly in all groups: from 44.7 % to 39.0 % after LSG, from 45.5 % to 36.5 % after LGCP and from 45.5 % to 38.9 % after RYGB.

### Glycemic control

Fasting glucose and glycated hemoglobin (HbA1c) levels decreased significantly in all surgical groups. Glucose levels dropped from 5.9 to 5.1 mmol/l after LSG, from 6.1 to 5.2 mmol/l after LGCP and from 7.9 to 5.4 mmol/l after RYGB. Similarly, HbA1c decreased significantly, reflecting improved long-term glucose regulation in all procedures.

### Lipid metabolism

Triglycerides decreased from 2.2 to 1.2 mmol/l after LSG, from 2.6 to 1.1 mmol/l after LGCP and from 2.4 to 1.3 mmol/l after RYGB. HDL levels increased significantly in LSG and LGCP patients. LDL cholesterol remained mostly unchanged, except for a slight, nonsignificant decrease in RYGB patients.

### Adipokines

Adiponectin levels increased significantly after all procedures, especially at 12 and 18 months. On the contrary, leptin concentrations decreased markedly, reflecting reduced fat mass and improved leptin sensitivity.

### ANGPTL proteins

Significant changes in ANGPTL4 levels were observed after LSG at 3 and 6 months, after LGCP at 3 and 12 months and after RYGB at 6 months. ANGPTL3 levels remained stable except for a significant increase at 18 months after LSG.

### Apolipoproteins

ApoD levels increased significantly only after LGCP at 6, 12 and 18 months. ApoE concentrations decreased significantly at early follow-up points in all surgeries but remained stable thereafter.

### FGF19 and FGF21

FGF19 levels increased significantly after LSG and LGCP, suggesting a potential role in improved glucose and lipid metabolism. No significant changes were observed after RYGB. On the contrary, FGF21 levels decreased significantly after all surgeries.

### Correlation analysis

**Fig. 1:**
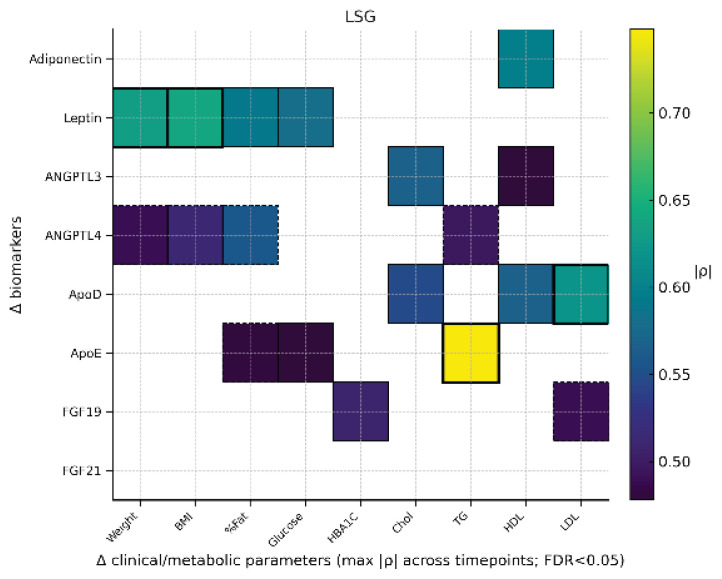
Heatmap LSG. Heatmap displays only FDR-adjusted significant pairs; color encodes |ρ|, solid borders indicate positive and dashed borders negative directionality; thicker borders denote |ρ|≥0.60.

**Fig. 2:**
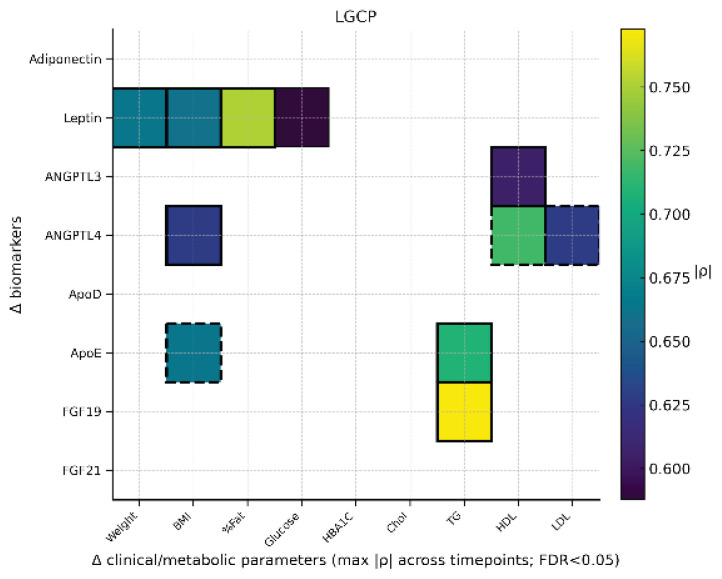
Heatmap LGCP. Heatmap displays only FDR-adjusted significant pairs; color encodes |ρ|, solid borders indicate positive and dashed borders negative directionality; thicker borders denote |ρ|≥0.60.

**Fig. 3:**
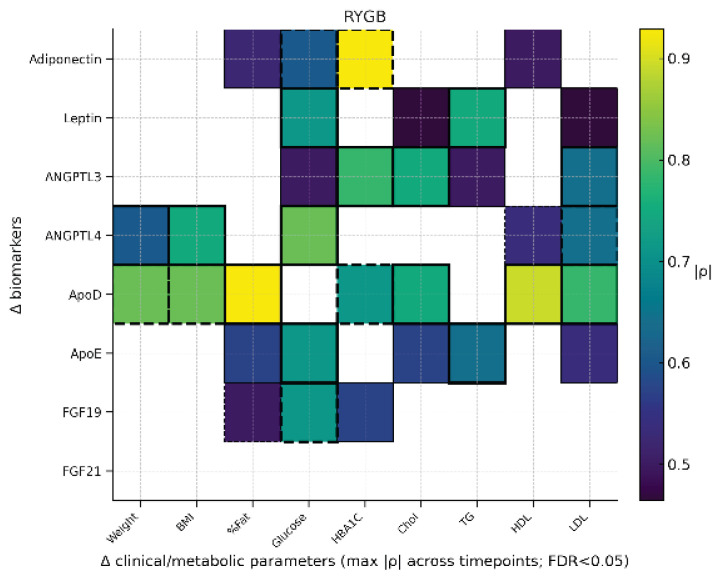
Heatmap RYGB. Heatmap displays only FDR-adjusted significant pairs; color encodes |ρ|, solid borders indicate positive and dashed borders negative directionality; thicker borders denote |ρ|≥0.60.

Across 3, 6, 12, and 18 months and within the LSG, LGCP, and RYGB groups, Spearman analyses (Δ defined as pre-post) revealed several robust associations (Figs 1–3). A correlation between ΔApoE and ΔTG after LSG may suggest ApoE’s role in modulating lipoprotein clearance and redistribution of triglyceride-rich particles during postoperative lipid metabolism adaptation. In LGCP, a consistent association emerged between ΔFGF19 and ΔTG, representing one of the most prominent lipid-related findings. In RYGB, the most pronounced (predominantly inverse) association was observed between ΔADI (ΔAdiponectin) and ΔHbA1c, aligning with improved glycemic control after surgery. Additional significant relationships (e.g., invol-ving leptin, apolipoproteins, and lipid profiles) were group-specific, of moderate-to-high magnitude, and showed less consistency across time points.

## Discussion

At present, bariatric operations are the most effective solution for obesity and to improve obesity-related comorbidities [2,3]. Our measured data demonstrate the endocrine effect of all three types of operations. Rapid weight reduction 3 months after operations is a consequence of dramatic changes in food intake and appetite, typical for these surgeries. Some significant differences in measured results were found among three procedures (LGCP, LSG, and RYGB).

One possible interpretation of the data is the impact of individual surgical procedures on endocrine interactions within selected biomarkers. In patients after RYGB, a strong inverse (negative) correlation was found between the change in adiponectin (ΔADI) levels and the change in HbA1c or glucose (ΔHbA1c/Glucose) levels. The improvement in glycemia is not only due to weight loss itself, but also to hormonal and metabolic changes (the so-called endocrine effect of bariatric surgery) [17]. A strong correlation was also found between ΔApoD and change in fat percentage. This suggests that ApoD may be involved in the regulation of fat accumulation or fat tissue remodeling. A possible mechanism is that higher adiposity leads to increased ApoD expression in fat cells as an adaptive response to increased lipid stress, oxidative stress, or a higher need for transport of hydrophobic lipid mediators (e.g., arachidonic acid). ApoD could thus act not only as a marker of fat quantity, but potentially also as a modulator of lipid metabolism, influencing fat storage or mobilization in adipocytes [18,19].

In patients after LGCP bariatric surgery, a strong inverse correlation was observed between the change in fibroblast growth factor 19 (ΔFGF19) levels and the change in triglyceride (ΔTG) levels. This correlation supports the role of the enterohepatic axis of the farnesoid X receptor (FXR) and FGF19 in the regulation of lipid metabolism after surgery, which modulates the flow of bile and nutrients [20].

After LSG bariatric surgery, a significant correlation was found between the change in apolipoprotein E (ΔApoE) levels and the change in triglyceride (ΔTG) levels. This correlation suggests that ApoE may play a role in regulating lipid metabolism after surgery. ApoE is key to lipid metabolism, particularly in the transport of triglycerides and cholesterol in lipoproteins. After bariatric surgery such as LSG, there are significant changes in the lipid profile, including a reduction in triglyceride levels. The increase in ApoE levels may be an adaptive response to these changes, facilitating lipid metabolism and improving the lipid profile [21].

An also interesting perspective on the impact of bariatric surgery on glucose and lipid metabolism is the interpretation based on biochemical markers. Regarding glucose metabolism, all types of surgery lead to a significant reduction in glucose and glycated hemoglobin concentrations, which is consistent with previously published results [22,23].

Among the parameters of lipid metabolism, approximately 60 % of patients with severe obesity who are candidates for surgical treatment for obesity have dyslipidemia [24]. Significant improvements in lipid and lipoprotein parameters after bariatric surgery occur early in the postoperative period, prior to weight loss, and persist throughout the follow-up [21]. The results of our study showed a similar trend. Regardless of the type of surgery, a significant reduction in triglycerides and an increase in HDL cholesterol were found. However, contrary to the literature, no significant changes in LDL cholesterol levels were observed in our study. ΔApoE associated with LDL/HDL reflects its key role in lipoprotein clearance *via* LDL receptor pathways and lipoprotein remodeling after surgery. ApoD is a component of HDL and is often associated with HDL function and CVD risk; therefore, HDL/LDL associations in our data indicate remodeling of HDL dynamics after weight loss and hormonal changes [25,26].

Our study also aimed to reveal the effects of the regulatory factors ANGPTL3 and ANGPTL4 on lipid metabolism. These factors are known to inhibit lipoprotein lipase and thereby controls TG cleavage and lipid redistribution. Significant changes in ANGPTL4 levels were observed after LSG at 3 and 6 months, after LGCP at 3 and 12 months and after RYGB at 6 months. No significant changes in ANGPTL3 levels were observed, except for a significant increase at 18 months after LSG. Our findings demonstrate a differential response of ANGPTL3 and ANGPTL4 following bariatric surgery. It suggest that ANGPTL4 is a sensitive marker of postoperative lipid metabolic adaptations. These results are consistent with previous studies indicating that ANGPTL4 plays a key role in regulating triglyceride metabolism by inhibiting lipoprotein lipase, and that its plasma concentration decreases in response to weight loss and improved insulin sensitivity [27,28]. In contrast, ANGPTL3 levels remained largely stable, with the exception of a delayed increase at 18 months after LSG. The unexpected increase in ANGPTL3 levels 18 months after LSG may reflect compensatory hepatic responses to altered lipid metabolism, including increased lipoprotein lipase activity and changes in hepatic fatty acid handling. Additionally, modifications in the gut-liver axis, such as altered bile acid profiles and microbiota composition, could influence ANGPTL3 expression [29]. The variation in ANGPTL3 responses between bariatric procedures suggests that surgical technique plays a key role in regulating this lipoprotein [28]. Overall, these data highlight the differential regulation of angiopoietin-like proteins after bariatric procedures and underscore the potential utility of ANGPTL4 as an early biomarker for postoperative improvements in lipid metabolism.

Adiponectin levels showed an increasing trend after all surgeries. A significant increase was found at 12 and 18 months after each of operations. This fact is not surprising; it is known that adiponectin levels steadily increase with time in parallel with weight loss [23]. A similar observation was made in cases of nonsurgical weight management [30].

Leptin serum levels were increased in obese patients compared to lean individuals due to leptin resistance in overweight and obese patients [31]. A significant decrease in leptin levels was found at every timepoint after all surgeries. This measurement is also in line with the available results in the literature [32].

FGF19 and FGF21 levels were both affected by bariatric surgeries. Some recent studies have suggested that FGF19 may have an important role in regulating glucose and energy metabolism [24]. Its potential to improve glucose control may be due in part to its effects on reducing body fat, promoting brown fat development, and increasing energy use [25]. It has been suggested that FGF19 may have a role to play in enhancing glucose effectiveness, which could mean that it helps glucose regulate its own uptake even when insulin levels are stable [26]. In humans, higher levels of FGF19 have been associated with lower amounts of visceral fat [24]. FGF19 is believed to promote better glucose regulation and overall metabolic balance through several pathways [13]. Some studies have suggested that RYGB can lead to significant increases in FGF19 levels [27]. These elevations are believed to be associated with improved glucose metabolism and could potentially contribute to the remission of type 2 diabetes mellitus (T2DM). In our study, there appeared to be an increased trend in FGF19 concentration in RYGB patients, although this did not reach statistical significance. However, there is evidence to suggest that patients have experienced a notable increase in FGF19 levels after LSG and LGCP surgery [28,29]. Our findings showed a significant increase in FGF19 levels at 12 and 18 months after LGCP, while in the LSG group a significant increase was seen at 6 and 18 months.

The delayed significant increase observed in our study at 18 months post-LGCP may reflect the time required for the enterohepatic signaling axis, particularly the FXR-FGF19 pathway, to fully activate and exert its metabolic effects. This pathway, influenced by elevated bile acids post-surgery, plays a crucial role in regulating glucose and lipid metabolism. The gradual elevation in FGF19 levels observed in our study suggests a progressive adaptation of this pathway following LGCP [20].

We also observed a strong correlation between ΔFGF19 and ΔTG after LGCP. This fits well with the FXR-FGF19-bile acids axis: FXR activation in the intestine increases FGF19, which suppresses bile acid synthesis (CYP7A1) and is thought to mediate liver-intestine cross-talk with effects on lipids and glucose. After bariatric surgery (especially RYGB/LSG), FGF19 is usually elevated and is associated with metabolic improvement; the link to TG in our data clinically supports this axis [33,34].

It is understood that FGF21 is a hormone that plays a key role in the regulation of energy metabolism, particularly in glucose and lipid homeostasis [14]. Its production is stimulated during nutritional stress, such as prolonged fasting, high-fat diets, or increased mitochondrial and endoplasmic reticulum stress [35]. It appears that the primary site of synthesis of FGF21 is the liver, although there is also evidence of its expression in adipose tissue, the pancreas, and the brain [36,37]. Research suggests that, unlike other bariatric surgeries, such as LSG, FGF21 levels remain stable after LGCP and RYGB [38]. Some studies show an increase in FGF21 levels within a short time after LSG surgery (till 6 months after surgery) [39]. This rise may be due to acute energy stress and metabolic resetting after surgery. It has been suggested that elevated FGF21 may be associated with weight loss and improved insulin sensitivity.

This was not the case in our cohort. We observed a decreasing trend in FGF21 levels following each type of surgery, with the reduction reaching statistical significance in many instances. Across all surgical procedures, FGF21 levels were significantly lower at 18 months postoperatively.

These findings highlight the complexity and heterogeneity of FGF21 responses following bariatric surgery. While some studies report an early postoperative rise in FGF21, likely reflecting acute energy stress and metabolic resetting [39], our data demonstrate a gradual decline over time, reaching significance at 18 months. This biphasic pattern – an initial transient increase followed by a longer-term decrease – may account for the variability observed between studies and across different surgical techniques [40]. Such differences could be influenced by patient characteristics, the type and extent of surgery, baseline metabolic status, and the timing of sample collection [38]. Overall, these results underscore the need for longitudinal monitoring of FGF21 and careful consideration of inter-individual variability when interpreting post-bariatric metabolic adaptations [39].

## Strengths of the Study

This study has several important strengths. It provides a comprehensive, prospective, and relatively long-term (18-month) evaluation of metabolic and hormonal changes following three distinct types of bariatric surgery, including both widely performed (LSG, RYGB) and less commonly studied (LGCP) procedures. This comparative approach offers valuable insights into the differential metabolic effects of various surgical techniques. Second, the study included a broad panel of biomarkers, encompassing adipokines, myokines, hepatokines, angiopoietin-like proteins, and apolipoproteins, allowing for a detailed assessment of the endocrine and metabolic adaptations following surgery. This multi-dimensional analysis extends beyond traditional anthropometric and biochemical outcomes commonly reported in bariatric surgery studies.

## Limitations

This study has several limitations. First, it is an observational cohort without randomization. The primary analyses rely on Δ-Δ associations (Δ = pre - post) summarized by Spearman correlation and complemented by robust ordinary least squares regression with HC3 covariance. Although we controlled the false discovery rate using the Benjamini-Hochberg procedure, causal inference cannot be made and residual confounding (e.g., changes in diet, physical activity, or concomitant medications) cannot be excluded.

Second, the sampling schedule did not capture the very early postoperative phase. Biomarkers were assessed at 3, 6, 12, and 18 months, but not within the first weeks to 2 months after surgery, when transient excursions – particularly for FGF21 and bile acid-related signaling – may occur. Our heat maps aggregate the maximum absolute Spearman ρ across the available postoperative time points for FDR-significant pairs; while this improves readability, it may obscure temporal dynamics and underrepresent time-specific patterns.

Third, subgroup sizes after stratification by surgical procedure (LSG, LGCP, RYGB) were modest, which reduces power, increases the risk of type II error, and limits between-procedure comparisons.

Fourth, the study was not designed for mechanistic dissection of the implicated pathways. We did not measure bile acid profiles or hepatic transport/synthesis markers (e.g., BSEP, CYP7A1) to directly interrogate the FXR-FGF19 enterohepatic axis, nor functional indices of the LPL-ANGPTL3/ANGPTL4 pathway (e.g., LPL activity, lipoprotein subfractions, or genetic variation). As a result, the observed relationships between FGF19 and triglycerides, or between ANGPTL3/ANGPTL4 and TG/HDL-C, should be interpreted as associative.

Finally, the single-center design and characteristics of our cohort may limit generalizability. Between-study heterogeneity is well recognized in bariatric populations – across procedures, baseline risk profiles, and assay platforms – and external validation in larger, more diverse cohorts will be important to confirm the biomarker-phenotype relationships reported here.

## Conclusions

All three bariatric procedures (LSG, LGCP, and RYGB) resulted in significant improvements in glucose metabolism and lipid profiles, including reductions in fasting glucose, triglycerides, and glycated hemoglobin, along with increased HDL cholesterol. Leptin levels decreased and adiponectin levels increased consistently across all surgical groups, reflecting favorable changes in adipose tissue function. These alterations may be associated with fluctuations in ANGPTL protein levels. In particular, FGF19 levels increased significantly after LSG and LGCP, supporting their roles in metabolic regulation, while FGF21 levels significantly decreased in all groups, suggesting different hormonal adaptations after surgery. Differences in fat loss patterns and biomarker responses among procedures highlight the complex and procedure-specific metabolic effects of bariatric surgery. More research is needed to elucidate the mechanisms driving the differential changes in FGF19, FGF21, ANGPTL and apolipoproteins.

## Figures and Tables

**Table 1:** Anthropometric parameter changes 3, 6, 12 and 18 months after LSG (n=48), LGCP (n=35) and RYGB surgery (n=12).

	*Parameter*	Pre-operative examination	3 months after surgery	6 months after surgery	12 months after surgery	18 months after surgery
*LSG*	*Weight [kg]*	121.7 ± 20.6	100.2 ± 17.7***	97.6 ± 19.0***	93.6 ± 20.1***	93.2 ± 20.5***

	*BMI [kg/m* *^2^* *]*	41.6 ± 4.9	34.8 ± 4.93***	33.5 ± 5.0***	31.8 ± 5.1***	32.1 ± 5.3***
	*Fat DXA [%]*	44.7 ± 5.3	42.4 ± 5.36***	40.4 ± 6.2***	38.3 ± 6.5***	39.0 ± 6.6***
	*Waist [cm]*	115.9 ± 13.1	103.5 ± 12.0***	99.8 ± 12.0***	96.0 ± 11.6***	97.1 ± 13.1***

*LGCP*	*Weight [kg]*	124.6 ± 21.4	107.8 ± 20.7***	103.2 ± 18.7***	99.7 ± 20.4***	94.5 ± 14.9***

	*BMI [kg/m* *^2^* *]*	42.2 ± 5.9	35.6 ± 6.1***	34.6 ± 5.3***	33.3 ± 6.0***	32.2 ± 5.3***
	*Fat DXA [%]*	45.5 ± 4.8	42.1 ± 5.9***	38.5 ± 6.3***	36.7 ± 7.3***	36.5 ± 8.9***
	*Waist [cm]*	118.2 ± 12.5	107.4 ± 13.3***	104.4 ± 15.1***	103.3 ± 15.7***	103.9 ± 16.0***

*RYGB*	*Weight [kg]*	131.3 ± 27.0	106.5 ± 25.1^**^	105.0 ± 20.4***	100.2 ± 19.5***	97.9 ± 17.8***

	*BMI [kg/m* *^2^* *]*	45.7 ± 7.4	36.9 ± 6.5^**^	36.5 ± 5.2***	34.8 ± 4.5***	34.0 ± 4.5***
	*Fat DXA [%]*	45.5 ± 6.5	42.2 ± 7.0^**^	40.8 ± 6.2***	38.1 ± 6.4***	38.9 ± 7.4***
	*Waist [cm]*	129.9 ± 15.0	116.3 ± 12.0^**^	111.3 ± 10.4^**^	110.6 ± 10.3^**^	111.3 ± 14.2^**^

Data are expressed as mean ± standard deviation.

* Comparison with pre-operative examination p<0.05.

*** Comparison with pre-operative examination p<0.001.

**Table 2:** Biochemical parameter changes 3, 6, 12 and 18 months after LSG surgery (n=48).

*Parameter*	Pre-operative examination	3 months after surgery	6 months after surgery	12 months after surgery	18 months after surgery
*Glucose [mmol/l]*	5.9 ± 1.3	5.2 ± 0.8***	5.2 ± 0.8*	5.1 ± 0.9***	5.1 ± 0.8***
*HbA1c [%]*	4.3 ± 1.0	3.7 ± 0.4***	3.7 ± 0.4***	3.7 ± 0.4***	3.5 ± 0.4***
*Cholesterol [mmol/l]*	5.0 ± 1.1	5.0 ± 1.2	4.9 ± 1.0	5.3 ± 1.1	5.0 ± 0.8
*HDL [mmol/l]*	1.1 ± 0.3	1.2 ± 0.3*	1.3 ± 0.3*	1.5 ± 0.3***	1.5 ± 0.4***
*LDL [mmol/l]*	3.0 ± 0.8	3.1 ± 0.9	3.1 ± 0.8	3.3 ± 0.9	3.1 ± 0.6
*TG [mmol/l]*	2.2 ± 1.1	1.5 ± 0.5***	1.2 ± 0.4***	1.3 ± 0.5***	1.2 ± 0.6***
*Adiponectin [mg/l]*	12.5 ± 6.2	15.6 ± 7.1	17.4 ± 10.7*	21.0 ± 9.3***	21.0 ± 11.5***
*Leptin [μg/l]*	43.6 ± 14.5	26.7 ± 18.2***	22.8 ± 15.7***	21.1 ± 13.7***	22.7 ± 17.7***
*ANGPTL3 [μg/l]*	240.7 ± 78.0	223.9 ± 78.0	260.3 ± 90.8	274.1 ± 69.3	318.3 ± 84.0***
*ANGPTL4 [μg/l]*	69.1 ± 40.6	81.7 ± 33.4*	80.5 ± 42.9*	67.0 ± 27.5	69.7 ± 30.4
*ApoD [mg/l]*	5.3 ± 3.8	6.7 ± 8.1	6.2 ± 4.6	6.0 ± 3.3	5.1 ± 1.4
*ApoE [mg/l]*	15.6 ± 7.0	11.8 ± 3.9*	11.6 ± 5.7^**^	14.8 ± 1.6	-
*FGF19 [ng/l]*	179.1 ± 113.5	226.3 ± 164.7	234.5 ± 163.1*	242.2 ± 230.2	314.0 ± 222.2*
*FGF21 [ng/l]*	172.8 ± 102.8	140.2 ± 111.9	102.5 ± 114.5	63.1 ± 55.5*	55.9 ± 42.4***

Data are expressed as mean ± standard deviation.

* Comparison with pre-operative examination p<0.05.

*** Comparison with pre-operative examination p<0.001.

**Table 3:** Biochemical parameter changes 3, 6, 12 and 18 months after LGCP surgery (n=35).

*Parameter*	Pre-operative examination	3 months after surgery	6 months after surgery	12 months after surgery	18 months after surgery
*Glucose [mmol/l]*	6.1 ± 1.7	4.97 ± 0.6^**^	5.2 ± 1.0***	5.1 ± 0.7***	5.2 ± 1.0*
*HbA1c [%]*	4.4 ± 1.1	3.71 ± 0.4^**^	3.9 ± 0.5^**^	3.6 ± 0.4***	3.6 ± 0.4***
*Cholesterol [mmol/l]*	4.9 ± 1.0	4.9 ± 0.9	4.8 ± 0.8	4.9 ± 0.9	5.0 ± 1.0
*HDL [mmol/l]*	1.1 ± 0.3	1.3 ± 0.3*	1.3 ± 0.3*	1.5 ± 0.3***	1.7 ± 0.5***
*LDL [mmol/l]*	3.0 ± 0.8	3.1 ± 0.8	3.0 ± 0.7	3.0 ± 0.7	2.9 ± 0.8
*TG [mmol/l]*	2.6 ± 1.9	1.3 ± 0.6***	1.3 ± 0.5***	1.1 ± 0.8***	1.1 ± 0.4***
*Adiponectin [mg/l]*	13.0 ± 7.3	17.6 ± 7.6	18.5 ± 10.0*	19.3 ± 12.0*	23.7 ± 16.0*
*Leptin [μg/l]*	41.7 ± 13.6	24.1 ± 15.0***	22.2 ± 15.7***	20.0 ± 15.2***	23.1 ± 19.4*
*ANGPTL3 [μg/l]*	246.4 ± 69.7	243.8 ± 66.0	263.3 ± 74.7	269.7 ± 82.7	301.2 ± 96.7
*ANGPTL4 [μg/l]*	69.9 ± 32.7	78.5 ± 29.4*	83.4 ± 39.7	68.6 ± 29.1*	70.3 ± 31.1
*ApoD [mg/l]*	3.4 ± 1.9	5.6 ± 7.3	5.1 ± 5.5^**^	5.2 ± 2.3***	5.2 ± 2.2*
*ApoE [mg/l]*	21.1 ± 8.5	10.9 ± 3.7^**^	11.1 ± 3.5***	11.5 ± 2.0***	-
*FGF19 [ng/l]*	148.0 ± 113.4	184.2 ± 105.4	207.2 ± 135.2	233.8 ± 164.3*	322.4 ± 186.4***
*FGF21 [ng/l]*	112.2 ± 118.0	101.2 ± 107.7	94.2 ± 106.9	77.6 ± 103.1	56.9 ± 89.6*

Data are expressed as mean ± standard deviation.

* Comparison with pre-operative examination p<0.05.

*** Comparison with pre-operative examination p<0.001.

**Table 4:** Biochemical parameter changes 3, 6, 12 and 18 months after RYGB surgery (n=12).

*Parameter*	Pre-operative examination	3 months after surgery	6 months after surgery	12 months after surgery	18 months after surgery
*Glucose [mmol/l]*	7.9 ± 2.6	6.2 ± 2.1	6.1 ± 1.9*	5.1 ± 1.0*	5.4 ± 1.2*
*HbA1c [%]*	5.9 ± 2.5	4.3 ± 0.9*	4.2 ± 0.9*	3.8 ± 0.8*	3.8 ± 1.0*
*Cholesterol [mmol/l]*	4.9 ± 0.8	4.0 ± 1.1	3.8 ± 1.0*	3.8 ± 0.8*	4.0 ± 1.1
*HDL [mmol/l]*	1.2 ± 0.4	1.2 ± 0.4	1.2 ± 0.3	1.3 ± 0.3	1.5 ± 0.5
*LDL [mmol/l]*	3.0 ± 0.5	2.3 ± 0.9	2.2 ± 0.8	2.2 ± 0.6*	2.3 ± 0.7
*TG [mmol/l]*	2.4 ± 0.8	1.3 ± 0.4*	1.3 ± 0.4*	1.3 ± 0.7***	1.3 ± 0.6***
*Adiponectin [mg/l]*	14.1 ± 8.6	15.8 ± 9.0*	17.9 ± 9.7*	21.7 ± 9.0*	23.4 ± 15.8*
*Leptin [μg/l]*	49.2 ± 10.1	21.3 ± 17.0*	20.9 ± 15.1*	21.4 ± 15.9*	22.1 ± 19.7***
*ANGPTL3 [μg/l]*	213.8 ± 61.0	197.2 ± 74.9	199.4 ± 74.8	255.6 ± 100.9	271.4 ± 53.9
*ANGPTL4 [μg/l]*	71.1 ± 32.7	89.7 ± 39.1	94.8 ± 39.6*	76.8 ± 30.1	57.2 ± 14.7
*ApoD [mg/l]*	3.7 ± 1.6	3.1 ± 0.9	3.4 ± 1.2	4.7 ± 2.5	3.9 ± 1.4
*ApoE [mg/l]*	22.0 ±7.0	10.7 ± 5.4*	10.7 ± 5.4^**^	10.0 ± 5.7*	-
*FGF19 [ng/l]*	183.1 ± 154.7	211.6 ± 164.0	277.6 ± 240.8	212.1 ± 147.3	276.7 ± 241.3
*FGF21 [ng/l]*	186.5 ± 64.4	120.3 ± 75.4^**^	102.0 ± 80.6*	56.0 ± 62.9^**^	41.9 ± 53.6***

Data are expressed as mean ± standard deviation.

* Comparison with pre-operative examination p<0.05.

*** Comparison with pre-operative examination p<0.001.
